# The effects of two early parenting interventions on child aggression and risk for violence in Brazil (The PIÁ Trial): protocol for a randomised controlled trial

**DOI:** 10.1186/s13063-019-3356-x

**Published:** 2019-05-02

**Authors:** Joseph Murray, Iná S. Santos, Andréa D. Bertoldi, Lynne Murray, Adriane Arteche, Luciana Tovo-Rodrigues, Suélen Cruz, Luciana Anselmi, Rafaela Martins, Elisa Altafim, Tâmara Biolo Soares, Maria Gabriela Andriotti, Andrea Gonzalez, Isabel Oliveira, Mariângela Freitas da Silveira, Peter Cooper

**Affiliations:** 1 Postgraduate Programme in Epidemiology, Federal University of Pelotas, Pelotas, RS Brazil; 2 University of Reading, Reading, UK; 3 Pontifícia Universidade Católica do Rio Grande do Sul, Porto Alegro, Brazil; 4 Postgraduate Programme in Mental Health, University of São Paulo, Ribeirão Preto, SP Brazil; 5 Instituto Cidade Segura, Porto Alegre, RS Brazil; 6 Psychiatry and Behavioural Neurosciences, McMaster University, Hamilton, ON Canada; 7 University of Cape Town, Cape Town, South Africa

**Keywords:** Child aggression, Parenting interventions, Child development, Violence prevention

## Abstract

**Background:**

Children in many low- and middle-income countries (LMICs) are at high risk for exposure to violence and later violent behaviour. The World Health Organization has declared an urgent need for the evaluation and implementation of low-cost parenting interventions in LMICs to prevent violence. Two areas of significant early risk are harsh parenting and poor child cognitive and socio-emotional development. Parenting interventions suitable for LMIC contexts have been developed targeting these risk factors and have been shown to have promising effects. However, their impact on child aggression, a key precursor of violence, has yet to be determined. The Pelotas Trial of Parenting Interventions for Aggression (PIÁ) has been designed to address this issue.

**Methods:**

We are conducting a randomised controlled trial to evaluate two early parenting interventions for mothers of children aged between 30 and 42 months in a Brazilian city. The first of these, dialogic book-sharing (DBS), aims to promote child cognitive and socio-emotional development; and the second, the ACT Raising Safe Kids Program (ACT), is designed to reduce harsh parenting. These interventions are being compared with a control group receiving neither intervention. Three hundred and sixty-nine families in a birth cohort are being randomly allocated to one of the three groups (DBS, ACT, Control). Facilitators deliver the interventions to groups of five to 10 mothers at weekly sessions for 8 weeks in DBS and 9 weeks in ACT. Independent assessments of parenting and child development are being made before the interventions, shortly afterwards, and at follow-up 6 months later. The primary outcome is child aggression, and the two main secondary outcomes are: (1) child cognitive and socio-emotional development and (2) harsh parenting. Longer-term outcomes will be investigated as the birth cohort is followed into late childhood, adolescence, and adulthood.

**Discussion:**

The Pelotas Trial of Parenting Interventions for Aggression (PIÁ) aims to evaluate the impact of two early parenting interventions on child aggression and several other key risk factors for the development of violence, including aspects of parenting and child cognition and socio-emotional functioning. The study is being carried out in a LMIC context where violence constitutes a major social and health burden. Since the two interventions are brief and, with modest levels of training, readily deliverable in LMIC settings, a demonstration that they benefit parenting and reduce risk factors for violence would be of major significance.

**Trial registration:**

Brazilian Ministry of Health Register of Clinical Trials, ID: RBR-2kwfsk. Registered on 6 June 2018.

**Electronic supplementary material:**

The online version of this article (10.1186/s13063-019-3356-x) contains supplementary material, which is available to authorized users.

## Background

The World Health Organization (WHO) has declared ‘violence a leading worldwide public health problem’ [1, 2]. In 2013, interpersonal violence (outside of combat situations) caused 405,000 deaths and 29.5 million injuries warranting medical attention worldwide [3]. Latin America has the highest regional homicide rate [4]. Globally, round half of all children are exposed to some form of violence each year [5, 6] and 30% of women experience lifetime intimate partner violence [7]. In Brazil, the most populous Latin American country, interpersonal violence, mainly between young males, is the second leading cause of years of life lost after heart disease [8], and its economic cost is estimated at 5% of annual GDP [9]. Non-fatal violent victimisation is associated with a range of mental health problems, sexually transmitted diseases, and risk behaviours linked with chronic disease [10–12]. Key international bodies therefore consider global prevention of violence to be a priority [6, 13]. For example, UN Sustainable Development Goals 5 and 16 require major reductions in violence by 2030. Notably, the biggest challenges are in high-violence LMIC contexts, where data are particularly scarce on the effectiveness of preventive interventions [14–16].

Early interventions that reduce risk factors for violence are potentially important public health prevention strategies [17]. Some evidence suggests that such an approach could be effective. Thus, randomised trials of intensive nurse home-visiting and preschool enrichment programmes in the USA have found reductions in child maltreatment [18] as well as in children’s own future crime perpetration and violence [19]. Cost-benefit analyses show that much of the large, long-term gains of such early intervention programmes are driven by crime reduction [20]. However, in LMIC settings, the elevated short-term costs of most existing programmes and their need for highly trained professionals make them impracticable, and, to date, there has been little interest in the application of such preventive strategies in LMICs.

However, brief, less expensive, programmes supporting parents without the need for highly specialised professionals are potentially affordable in LMICs, and might have large benefits for children residing in impoverished environments [21, 22]. Although randomised controlled trials (RCTs) of parenting interventions have shown promising results in HICs [19, 23–26], few trials have been conducted in LMICs. A systematic review located only 12 such trials in LMICs by 2013 [27] with just two demonstrating adequate power and low risk of bias – and neither examined child behavioural outcomes.

The WHO has declared an urgent need for the evaluation and implementation of low-cost parenting interventions in LMIC contexts to prevent violence [28]. Several early family and personal factors are associated with increased risk for children’s persistent aggression – a key precursor of later violence perpetration [29]. Parenting programmes potentially could reduce children’s risk for developing persistent child aggression in two key ways. The first is by promoting parenting that provides good cognitive support to children (improving child learning and school readiness); and the second is reducing harsh and abusive parenting. There is robust evidence that interventions that help parents support their children’s cognitive development can be effective [30]. There is also evidence that parents can be helped to reduce harsh and abusive parenting [18]. The problem for LMICs is that parenting interventions that have been shown to be effective tend to be specialist and long term, which makes them, as noted, unaffordable in LMIC contexts. It is critical to the agenda of scale-up in LMIC settings that interventions are developed and evaluated that are affordable and deliverable by non-specialist personnel.

### Current trial

The Pelotas Trial of Parenting Interventions for Aggression (The PIÁ Trial) aims to evaluate the efficacy of two brief, parent-training programmes for reducing early child aggression. The study is being run in the city of Pelotas in southern Brazil, a LMIC. The trial is evaluating two low-cost, manualised parent-training programmes. These are: (1) a ‘dialogic book-sharing programme’ (DBS) that aims to improve child cognition and social understanding [31–33], and (2) ‘ACT: Raising Safe Kids program’ (ACT), which aims to reduce harsh parenting and child maltreatment [34]. The two interventions therefore target both sides of the individual and parenting risks highlighted above, putatively linking adverse environments to persistent child aggression. A three-arm RCT is being used to evaluate the impact of the two programmes. Interventions are being provided by local government personnel (i.e. primary care workers for DBS and school education coordinators for ACT) whom our team has trained as facilitators. The population participating in the trial is a high-risk subset – in terms of poverty and child aggression – of an ongoing birth cohort study, the 2015 Pelotas Birth Cohort Study [35]. Independent assessments are being made of child aggression, as well as the two key risk factors, child cognition and harsh parenting. Additional assessments are being made of broader parenting practices and child developmental progress. Assessments are being made on three occasions: before the intervention (when children are aged between 30 and 42 months), shortly following the intervention, and at a 6-month follow-up when the children are aged 4 years.

## Methods

### Study design

The study is a three-arm RCT. The three arms are:

1. Dialogic book-sharing (DBS) – a parenting intervention designed to promote sensitive and supportive interactions with children over picture books with the aim of improving child cognitive development and social understanding
2. ACT: Raising Safe Kids Program (ACT) – a parenting intervention designed to reduce harsh parenting; and
3. Control group – this group receives no intervention input from the research team, but continues to receive the standard support services available to the community from which the sample is drawn

### Hypotheses

The study has three hypotheses:

1. Compared to children of families who receive no intervention (the Control group), the children in families receiving DBS will show less aggression at follow-up, and they will perform better on measures of language, executive function, attention, and empathy/emotion understanding, but the parents will not show less harsh and abusive parenting
2. Compared to children of Control group families, the children of families receiving ACT will show less aggression at follow-up and their parents will show less harsh and abusive parenting and less favourable attitudes about corporal punishment, but the children will not perform better on measures of language, executive function, attention, and empathy/emotion understanding
3. For both the DBS and ACT groups, children and parents will show less stress at follow-up, and parents will show more positive parenting

### Collaboration and study setting

The study is being conducted in the city of Pelotas in southern Brazil from the Centre for Epidemiological Research at the Federal University of Pelotas. Four population-based, birth cohort studies are being run by the Federal University of Pelotas, including about 20,000 children born in 1982, 1993, 2004 and 2015 [35–38] and repeated follow-ups through childhood, adolescence and early adulthood. The current study is nested within the 2015 cohort with a view to monitor the impact of the interventions through the life-course. The assessments of the families are being conducted by trained assessors at the Research Centre where all assessments of the Pelotas cohorts are routinely conducted. The Pelotas Municipal Government is a collaborator on the trial and their staff are delivering the interventions in nursery and primary school facilities, with training and supervision by the research team. The Municipal Government is supporting the trial within a broader initiative called ‘Pacto Pelotas Pela Paz’, with a view to implementing evidence-based interventions to reduce violence in the city.

### Sample and eligibility criteria

The trial is embedded within an ongoing birth cohort study of 4275 children born in the city of Pelotas, southern Brazil (the 2015 Pelotas Birth Cohort Study) [35]. For The PIÁ Trial, about 20% of children in the cohort were first identified as potentially eligible for the study based on data collected previously with mothers when children were aged 2 years. Eligible children were first defined as high-risk on the basis of low family income when children were aged 2 years (bottom 30% of the sample). Children who were rated as very low on physical aggression by their mothers at age 2 years (0 or 1 on a 6-point scale from the ELDEQ study [39]; 31% of cohort children) were excluded from the trial, as were children who revealed signs of serious developmental delay (i.e. 10% with lowest scores on the INTER-NDA assessment at age 2 years [40]). Families are also being excluded if the child or mother has a condition preventing participation in the interventions, such as significant visual or auditory impairment, if the child has a live twin, or if they do not live within the Pelotas municipal urban boundary where the interventions are being delivered. Seven hundred and seventy-three children from the cohort met criteria as potential participants in The PIÁ Trial. The final sample is then being recruited by the research team contacting the families to confirm that the mother is currently responsible for the child (cares for the child at least 4 days a week), to explain the purposes of the study, to establish the mother’s availability to participate in the interventions if she were selected, and to invite mothers to participate in The PIÁ Trial. The children are 30 to 42 months at baseline assessment.

### Sample size

Sample size has been calculated based on a projected effect size for each intervention of mid-range, moderate magnitude (*d* = 0.45) for the primary outcome at 6-month follow-up, based on findings of well-conducted previous trials of parenting interventions for child behavioural disturbance [23, 41–43]. With alpha at 0.025 (due to two pair-wise comparisons (i.e. between DBS and Control, and between ACT and Control), and beta at 0.20, each of the three trial arms requires a minimum of 104 participants (allowing for 10% attrition to the 6-month follow-up). To allow for identification of potentially smaller effects at 6 months, and continued follow-up of the trial participants in later phases of development, a sample size of 369 is being recruited in total.

### Recruitment and randomisation

Having identified the eligible sample, each family is being contacted by a recruiter in their own home. The study is explained to the mother who is then invited to take part.

**Fig. 1:**
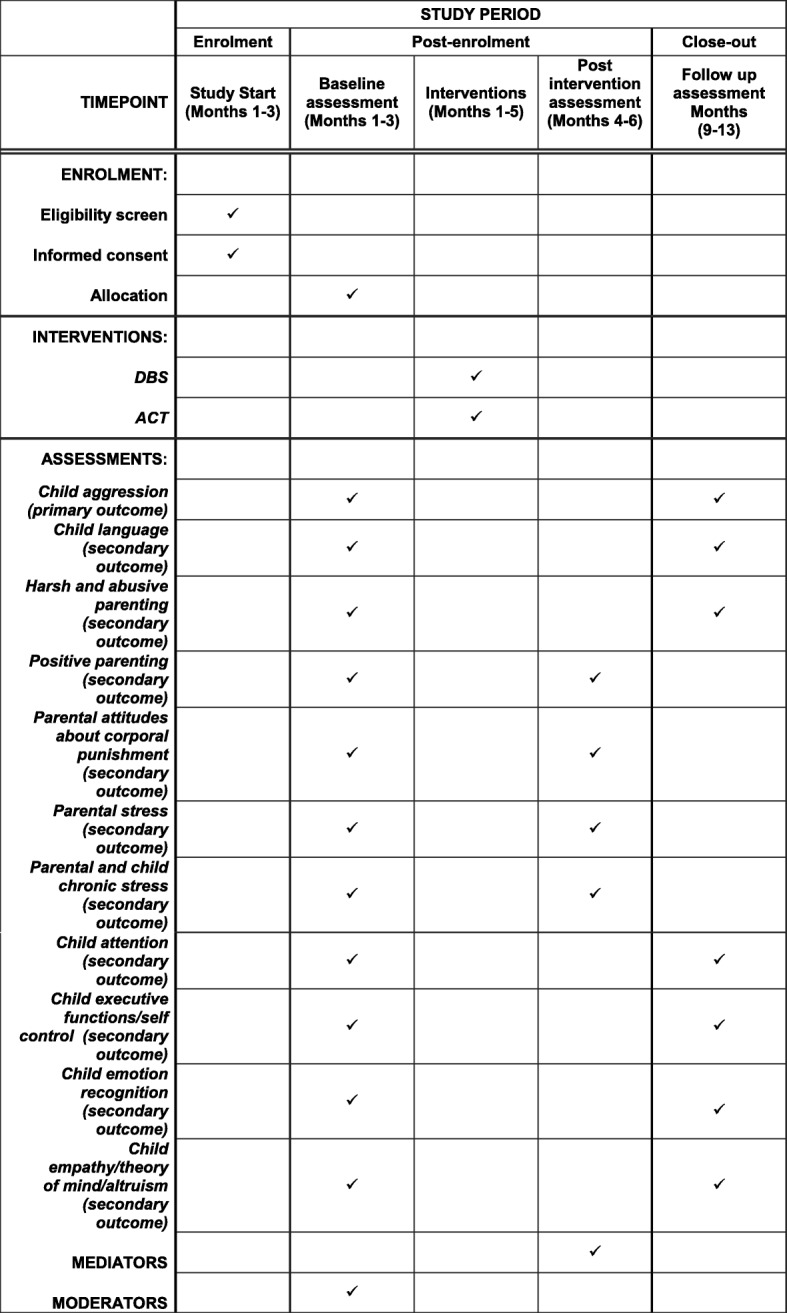
Schedule of enrolment, interventions, and assessments

From the 2015 cohort, 770 children met all the initial inclusion criteria above before being contacted in their homes. This sample was divided into 11 localities, each comprising approximately 70 families. Within each area, families are being invited to participate in the study (after confirming eligibility) in order of proximity to the intervention centre. Then, following consent and baseline evaluation, mother-child pairs are being randomised to one of the three study groups. This is being effected centrally (i.e. from the Federal University of Pelotas Centre for Epidemiological Research), minimising for child age, sex, level of harsh parenting at age 2 years, and child aggression at age 2 years (all binary variables). This process is being repeated serially across the 11 areas. The interventions are being delivered within public nursery/primary schools within each recruitment area. Assessments are being made at the Federal University of Pelotas Centre for Epidemiological Research (Fig. 1).

### The interventions

#### Intervention 1. Dialogic book-sharing (DBS: Mikhulu Trust/World Health Organization)

The WHO Violence and Injuries Prevention Unit recently assembled a suite of parenting interventions aiming to reduce risk factors for youth violence in LMICs. One of these is the DBS programme developed by the Mikhulu Trust. Central features of this intervention are, in the course of sharing a picture book with a child, becoming aware of the child’s focus of interest, responding sensitively, and engaging in reciprocal exchanges with the child. The training is delivered to families at weekly meetings over 8 weeks to small groups of parents in 90-minute sessions. Training in DBS is readily culturally transportable, and trials in both HICs [44] and LMICs [32, 33] have shown that it has medium-large effects on child language outcomes. Indeed, a trial in South Africa found large positive benefits to both child language and attention, and increased parental sensitivity (at least *d* = 0.78) [31–33], and a recent trial of DBS in Brazil similarly found medium-sized benefits to child cognition [45]. While these variables are known to be key protective factors against child aggression, no LMIC study has yet determined whether changing these cognitive factors is associated with reduced later child aggression. This will be tested in The PIÁ Trial.

#### Intervention 2: ACT Raising Safe Kids Program (ACT: Violence Prevention Office, American Psychological Association – APA)

The ACT Raising Safe Kids Program was developed by the APA as a non-profit, low-cost intervention with high cultural adaptability [34]. It consists of nine group-based sessions, delivered weekly in 2-hour sessions, in which parents are given information (through interactive activities, as well as slides and videos) on child development, strategies for emotion and behaviour regulation, positive communication, problem-solving techniques and guidance in how to raise children free of violence. Based on trials of ACT in HICs [46, 47] which found reductions in both harsh parenting and child conduct problems [48–50], the WHO recommends ACT as potentially appropriate for LMIC settings [51]. A before-after study [52, 53] examining a Brazilian version of ACT [54] found positive change in parenting and child behaviour, and that it was culturally acceptable across different socio-economic groups. Tables 1 and 2 summarise the two study interventions.

**Table 1:** Dialogic book-sharing (DBS) Book-sharing intervention session content

Session	Session content
1	Introduction to Book-sharing (using ‘Handa’s Surprise’ by Eileen Browne) The benefits to child development of book-sharing are explained, and the importance of establishing a book-sharing routine stressed. Basic principles of dialogic reading are outlined, including following the child’s lead, as well as techniques such as pointing and naming, and asking ‘who/what/where’ questions to engage the child and encourage a dialogue
2	Elaborating and Linking (using ‘Little Helpers’ by Lynne Murray, Peter Cooper, and Lyn Gilbert) Picking up on the child’s focus of interest and elaborating on it. Making links between the book content and the child’s own experience. Making links between different elements of the book, and their relation to the overall book narrative
3	Numeracy and Comparisons (using ‘Handa’s Hen’ by Eileen Browne) Introducing the idea of counting and comparative concepts (e.g. more, less, highest, smallest), and category inclusion and exclusion
4	Talking about Feelings (using ‘Hug’ by Jez Alborough) Talking about the feelings of the book characters. Naming feelings and contextualising them. Linking the book characters’ feelings to the child’s own emotional experience
5	Talking about Intentions (using ‘Harry the Dirty Dog’ by Gene Zion and Margaret Bloy Graham) Discussing why characters feel the way they do, asking what characters are thinking and intending, encouraging the child to be curious about what will come next in the story
6	Talking about Perspectives (using ‘Harry by the Sea’ by Gene Zion and Margaret Bloy Graham) Helping the child understand that different people can see things differently, know different things, and feel differently about things
7	Relationships (using ‘The Wrong Side of the Bed’ by Edward Ardizzone) Discussing family relationships, including conflict and resolution
8	Review session Recapitulation of key principles of book-sharing and discussion about how participants will take the book-sharing ahead in their day-to-day lives

**Table 2:** ACT Raising Safe Kids Program intervention session content

Session	Session content
1	Pre Meeting: Motivation and Behavioural Changes The benefits and objectives of the ACT program are explained and the rules of the group meetings are established. Parents are encouraged to think and discuss the dreams they have for their children
2	Learning Child Development and Understanding Children’s Behaviour Helping parents/caregivers learn basic elements of child development and how to respond appropriately to their children’s behaviour
3	Young Children’s Exposure to Violence Helping parents understand how children may be exposed to violence and the consequences it will have on their lives
4	Understanding and Controlling Parents/Adults’ Anger Helping parents learn to control and deal with anger
5	Understanding and Helping Angry Children Helping parents understand children’s feelings of anger, and learn how to teach them to control their feelings
6	Children and Electronic Media Helping parents understand the impact of electronic media on their children’s behaviour, and show them some options on how to reduce children’s exposure to violence
7	Discipline and Parenting Style Helping parents understand that the way they raise their children has an impact on their lifelong behaviour
8	Discipline for Positive Behaviours Teaching parents how to prevent difficult behaviours and how to use positive ways of disciplining children
9	Parents as Teachers, Protectors and Advocates at Home and in the Community Helping participants understand what they have learned from the ACT program, and that it is already helping them to make the dreams they have for their children come true. Encourage participants to use at home and in the community the tools they have learned and reinforce in parents their role as teachers, protectors and advocates for their children

### Training and supervision of interventions

The Pelotas City Government is implementing the two interventions under the supervision of the research team. There are 11 DBS facilitators and 14 ACT facilitators working on The PIÁ Trial. Community workers from the State Primary Care for Children Programme in Pelotas (Primeira Infância Melhor) are delivering the DBS intervention (DBS). Education coordinators and social workers are delivering the ACT intervention.

Training in DBS has been provided to these community workers by David Jeffery of the Mikhulu Trust (www.mikhulutrust.org) over a 5-day course, supported by TBS and MGA who were trained by PJC and LM. Training in ACT was provided through an in-person training workshop given by EA, a postdoctoral psychologist and ACT master trainer certified by the Violence Prevention Office of the APA. The ACT facilitators received further supervision and feedback by the ACT master trainer by distance (through video-taped sessions, a Google class room, and Skype discussions) and in-person by SC, a postdoctoral psychologist with clinical and child development experience. Both sets of training were conducted early in 2018, which allowed the intervention facilitators some months to practise the intervention techniques before their implementation in July 2018. During the implementation phase, TBS and MGA provide weekly supervision to the DBS facilitators and SC provides weekly supervision to the ACT facilitators with support from EA. A member of the research team is observing a random sample of 10% of group sessions and rating them for content to determine fidelity.

### Data collection

#### Data collector training

Ten experienced data collectors were trained over a 1-month period by senior psychologists (SC, LA, and AA) in the child and caregiver assessments specific to The PIÁ Trial, and followed a data collection manual developed by SC, LA, AA, and RM. This was done at the Federal University of Pelotas Centre for Epidemiological Research. Close ongoing supervision is being provided by LA. During the three assessment waves, regular checks are made through examination of the data, to ensure fidelity of assessment administration. All data collectors are familiar with consent and referral procedures, as well as how to consider potentially sensitive topics with caregivers during the assessment.

#### Procedures

All mother/child pairs are assessed on three occasions: at baseline, 4 weeks following the intervention, and 6 months post intervention. For the baseline assessment, mothers are contacted by study recruiters and the study is explained to them. It is emphasised that participation is entirely voluntary and that non-participation carries no consequences. A suitable time for them to come to the Research Centre for assessment by the data collector is arranged. On arrival at the assessment session, consent is explained again and caregivers provide written consent for both themselves and their child. Assessments take 2 h and 20 min, on average. They comprise specific assessments of the child (e.g. language assessment), questionnaires completed by the caregiver (e.g. child behaviour), and filming the caregiver and child in interactive tasks (e.g. during book-sharing). There are breaks for refreshment and, if the child shows signs of tiredness or distress, the session is interrupted or, if necessary, terminated. Participants are given a small gratuity for contributing their time to the study. Similar procedures are followed for the subsequent two assessment waves. To prevent assessor bias, assessments of children and caregivers are being carried out blind to group allocation, with the at random allocation being conducted separately at the end of the baseline assessment. Participants are explicitly asked to not reveal their allocation to the data collectors in follow-up assessments. All coding of video material will be made blind to allocation.

#### Retention

Provisions have been put in place to maximise participant retention. This includes texts and phone calls to remind participants of scheduled assessments and sessions, fridge magnets to remind participants of the scheduled time, snacks, travel funds, and small gifts as well as a raffle for each study arm with the prize of a tablet. To increase adherence in the interventions, the mothers are: (1) shown videos about the benefits of the interventions, spoken by local mothers who had previously completed them, (2) taken by van to attend the first session and other sessions where their home is far from the intervention centre, (3) given travel funds for attendance at each session, (4) given snacks and a small gift for the children, and (5) provided with childcare during the sessions.

## Outcomes

### Assessments

Detailed assessments of the trial participants are being made at baseline and will be conducted on two occasions post intervention: 4 weeks following the end of the interventions and then at a the 6-month follow-up (see Table 3 below).

**Table 3:** Study outcomes and measures

Outcome	Concept	Measures	Baseline (months 1–3)	Post intervention (months 4–6)	Follow-up (months 9–13)
Child aggression (primary outcome)	Child aggression	Aggression sub-scale of the Child Behaviour Checklist	✓		✓
		Items on aggression from the ELDEQ Study Questionnaire	✓		✓
		Observation: Don’t touch’ and ‘Clean Up’ tasks	✓		✓
		Observation: LabTab, ‘Don’t touch’ and ‘Clean Up’ tasks	✓		✓
Child language (main secondary outcome)	Expressive language	Teste de Vocabulário Expressivo	✓		✓
	Receptive language	Teste de Vocabulário Receptivo	✓		✓
Parenting (main secondary outcome)	Harsh and abusive parenting	PAFAS Questionnaire	✓		✓
		Juvenile Victimisation Questionnaire	✓		✓
		Observation: Don’t touch’ and ‘Clean Up’ tasks	✓		✓
		Searches of child protection service records			✓
Parenting (secondary outcomes)	Positive parenting	PAFAS Questionnaire	✓	✓	
		Videotaped sensitivity and reciprocity during book-sharing and free-play parent-child interactions	✓	✓	
	Parental attitudes about corporal punishment	Deater-Deckard study Questionnaire	✓	✓	
Stress (secondary outcomes)	Parental stress	Perceived Stress Scale	✓	✓	
		Pelotas questions on parenting stress	✓	✓	
	Parental and child chronic stress	Cortisol from hair samples	✓		✓
Child development (secondary outcomes)	Child attention	Strengths and Difficulties Questionnaire	✓		✓
		Card Sort task from the Early Years Toolbox			✓
	Child executive functions/self-control	Go no Go task from the Early Years Toolbox	✓		✓
		Block Design task	✓		✓
	Child emotion recognition	Denham’s puppet task	✓		✓
	Child empathy/theory of mind/altruism	Em-Que Questionnaire	✓		✓
		Help task	✓		✓
		Dictator Game			✓
		Triangle task	✓		✓
		Sally-Anne task			✓

The primary outcome is child aggression at the 6-month follow-up assessment, measured by parental report, and direct observation. The parent report measures include two questionnaires: the Aggression sub-scale of the Child Behavior Checklist (CBCL) [55] and items on aggression from the ELDEQ Study Questionnaire [39]. Three observational measures are being used: child response to a frustration task (from the *Laboratory Temperament Assessment Battery* for preschool children, www.uta.edu/faculty/jgagne/labtab), and child behaviour during ‘Don’t touch’ and ‘Clean Up’ tasks [56, 57]. The multiple measures of aggression will be combined into at least one latent variable for analysis of the primary outcome, and the independent trial statistician will decide if a single variable or multiple latent variables are required (for example, one for observed aggression and one for reported aggression).

The two main secondary outcomes will be measured at the 6-month follow-up. All relevant measures are being administered to the whole trial sample, but effects are hypothesised to be specific to DBS or ACT, as outlined above:

1. Child language will be assessed using the Test of Receptive and Expressive Vocabulary (Teste de Vocabulário Auditivo e Teste de Vocabulário Expressivo http://memnon.com.br/produto/teste-de-vocabulario-auditivo-e-teste-de-vocabulario-expressivo/)
2. Harsh and abusive parenting will be assessed by parent self-report, using the PAFAS Questionnaire [58], the Juvenile Victimisation Questionnaire (http://www.unh.edu/ccrc/jvq/index_new.html), by direct observation during the ‘Don’t touch’ and ‘Clean Up’ tasks [56, 57], and by searches of child protection service records

Additional secondary outcomes will be measured at the 4-week post-intervention assessment (#1–4 below) and at the 6-month follow-up (#5–8).

1. Positive parenting will be assessed using the PAFAS Questionnaire, and videotaped sensitivity and reciprocity during book-sharing and free-play parent-child interactions (as successfully used in previous book-sharing trials) [31–33]
2. Parental attitudes about corporal punishment will be assessed using by the Deater-Deckard Study Questionnaire [59]
3. Parental stress will be assessed using the Perceived Stress Scale [60] and Pelotas questions on parenting stress
4. Parental and child chronic stress will be assessed by cortisol from hair samples [61–65]
5. Child attention will be assessed using the Strengths and Difficulties Questionnaire [66] and the Card Sort task from the Early Years Toolbox [67]
6. Child executive functions/self-control will be assessed using the Go no Go task from the Early Years Toolbox [67], the Block Design task, and assessor ratings
7. Child emotion recognition will be assessed using Denham’s puppet task [68]
8. Child empathy/theory of mind/altruism will be assessed using the Em-Que Parent Questionnaire measure [69], the Help task [70], the Dictator Game [71], and the Sally-Anne task [72]

All the outcome measures specified above are being taken at baseline, except the Dictator Game measure of altruism and the Card Sort Game, which were judged to be less amenable to repeat application over a short period of time, and the Sally-Anne task (for empathy, the Triangle task [73] is being used at baseline, but concerns about how well it is functioning require an additional measure for follow-up)

### Potential moderators

The following variables will be examined as potential moderators: parental education, parental mental health, domestic violence, and maternal and child stress, maternal harsh parenting, number of siblings, and child sex, age, and aggression.

### Data management

Participants are being assured of the confidentiality and anonymity of their data. Data are being anonymised by using ID codes which are kept in secure storage on Federal University of Pelotas premises, with individuals’ personal, identifiable details kept separate from all other information. The anonymised electronic data will be archived at the Federal University of Pelotas, Centre for Epidemiological Research data storage and archive division (under the supervision of Cauane Blumenberg, Research Data Manager). Data will be made available to the academic community via requests being sent to the Pelotas Cohorts Publications’ Committee. Sensitive data will be stored in the archive under a restricted access setting, accessible to the data depositor and archive administrative staff only.

## Data analysis

Data analysis will be completed by a designated statistician, Merryn Vossey from the Oxford University Department of Primary Care Medicine, who will work independent from study investigators. Group baseline differences will be investigated including socio-demographic data, such as child sex, and household factors (e.g. income, relationship status), and study outcomes.

The primary and secondary outcomes will be analysed using linear mixed models, which can account for repeated assessments within individuals (for outcomes measured at multiple time-points). Intervention effects will be assessed at post intervention and follow-up and will be adjusted for child’s age, sex, and baseline scores (where applicable). Further socio-demographic factors may also be investigated as covariates. If the necessary assumptions of the models do not hold, suitable alternative models will be fitted. Intention-to-treat analysis will be used to examine intervention effects. Sensitivity analyses will examine if intervention effects maintain for measures that are not dependent on parent report, which may be biased because parents are not blind to the interventions.

The amount and pattern of missing data will be examined and will be addressed using multiple imputation where appropriate. Due to the multiplicity of comparisons, caution will be used in interpreting results of secondary outcome comparisons. No single *p* value will be interpreted in isolation and all findings will be considered together to obtain the full picture of the intervention effects on the different outcome measures.

### Mediator analyses

Mediator analyses will aim to identify active components of the interventions and elucidate the pathways to change. To this end, the following question will be examined: whether the impact of the interventions on child aggression is mediated by improvements in child cognition and by reductions in harsh parenting.

### Moderator analyses

Moderator analyses will be conducted to investigate whether certain groups respond differently to the interventions. In addition to the potential mediators listed above, we will examine the impact of number of intervention sessions attended. Potential mediators and moderators of the intervention will be examined using mixed linear models or structural equation modelling, as appropriate.

### Trial monitoring

#### Trial Steering Committee

An independent Trial Steering Committee (TSC) is monitoring the progress of the trial and advises the research team on matters arising during the course of the study. The PI (JM) consults with the TSC Chair once a month and the TSC meets biannually. The TSC is chaired by Prof Cathy Ward (Chair), Department of Psychology, University of Cape Town. Other external academic representation is provided by Prof Manuel Eisner, Institute of Criminology, University of Cambridge; Prof Pasco Fearon, Division of Psychology and Language Sciences, University College London, and Dr. Christian Kieling, Department of Psychiatry, Federal University of Rio Grande do Sul. Marilia Mesenburg, a mother of a child in 2015 Pelotas Birth Cohort Study (not selected for the trial) represents the local Pelotas community. TSC members from The PIÁ Trial study team are JM and IS.

## Discussion

The PIÁ Trial is an evaluation of two parenting interventions, both with the potential to reduce risk for later offspring violence. The DBS intervention targets child cognitive function/social understanding, which is implicated in the development of persistent child aggression, itself a strong predictor of later violence. The ACT programme targets harsh parenting and maltreatment, also associated with child aggression and later violence. The interventions are being delivered to mothers of 30–42-month old children in the Brazilian city of Pelotas, a city with a high rate of socio-economic disadvantage and a very high level of violence. The interventions are being delivered by trained facilitators, during weekly sessions over 8–9 weeks, to small groups of mothers. The primary outcome of The PIÁ Trial is child aggression. The two main secondary outcomes are child language and harsh parenting. A number of other assessments are being made, both of parenting and of child developmental progress. Parental reports of child behaviour may be biased because parents are, of course, not blind to their intervention status. However, The PIÁ Trial also includes observational measures of child behavior and parenting, direct tests with children, as well as external data sources (records), reducing this bias.

A major strength of the trial is that it is embedded within a birth cohort study, and the intention is to follow-up the cohort, including the trial participants, over many years. Indeed, The PIÁ Trial will be one of the few studies of early parenting interventions aiming to assess offspring outcomes into adulthood, and perhaps the only early parenting trial aiming to investigate long-term impact on aggression through the life-course [74].

### Outcomes, outputs, and dissemination

Following receipt of the trial statistical report, we will disseminate the study findings in several ways. We will publish them in peer reviewed academic journals and in relevant professional journals. We will produce a summary of the project’s objectives, methodologies, and key findings, together with recommendations for policy and practice, which will appear on the Federal University of Pelotas University and Instituto Cidade Segura websites. We will also write a briefing paper for distribution to the local government of Pelotas and local and regional press.

## Trial status

At the point of submitting this manuscript to the journal (16 August 2018), 304 out of the final 369 participants in the sample had been recruited. This paper represents version one of the protocol (Additional file 1).

## Additional file

Additional file 1: Standard Protocol Items: Recommendations for Interventional Trials (SPIRIT) 2013 Checklist: recommended items to address in a clinical trial protocol and related documents*. (DOC 119 kb)

## Abbreviations

ACT: ACT Raising Safe Kids Program
APA: American Psychological Association
DBS: Dialogic book-sharing
HIC: High-income country
LMIC: Low- and middle-income country
PI: Principal investigator
PIÁ: Pelotas Trial of Parenting Interventions for Aggression
RCT: Randomised controlled trial
WHO: World Health Organization

## References

[1] World Health Organization (2002). World report on violence and health.

[2] World Health Organization (2014). Strengthening the role of the health system in addressing violence, in particular against women and girls, and against children (Resolution WHA67.15).

[3] Haagsma JA, Graetz N, Bolliger I, Naghavi M, Higashi H, Mullany EC, Abera SF, Abraham JP, Adofo K, Alsharif U (2016). The global burden of injury: incidence, mortality, disability-adjusted life years and time trends from the Global Burden of Disease study 2013. Inj Prev.

[4] Chioda L (2017). Stop the violence in Latin America: a look at prevention from cradle to adulthood.

[5] Hillis S, Mercy J, Amobi A, Kress H (2016). Global prevalence of past-year violence against children: a systematic review and minimum estimates. Pediatrics.

[6] United Nations Children’s Fund (2014). Hidden in plain sight: a statistical analysis of violence against children.

[7] Devries KM, Mak JYT, García-Moreno C, Petzold M, Child JC, Falder G, Lim S, Bacchus LJ, Engell RE, Rosenfeld L (2013). The global prevalence of intimate partner violence against women. Science.

[8] Global Burden of Disease 2015 Data. http://www.healthdata.org/gbd/data.

[9] Cerqueira DRC, Carvalho AXY, Lobão W, Rodrigues RI. Análise dos custos e conseqüências da violência no Brasil [Analysis of the costs and consequences of violence in Brazil]. Brasilia, DF, Brazil: Institute de Pesquisa EconômiCa Aplicada; 2007.

[10] Gilbert R, Widom CS, Browne K, Fergusson D, Webb E, Janson S (2009). Burden and consequences of child maltreatment in high-income countries. Lancet.

[11] Matzopoulos R, Bowman B, Butchart A, Mercy JA (2008). The impact of violence on health in low- to middle-income countries. Int J Inj Control Saf Promot.

[12] Ellsberg M, Jansen HAFM, Heise L, Watts CH, Garcia-Moreno C. Intimate partner violence and women's physical and mental health in the WHO multi-country study on women's health and domestic violence: an observational study. Lancet. 2008;371:1165–72. 10.1016/S0140-6736(08)60522-X 18395577

[13] World Health Organization. Global Status Report on Violence Prevention 2014. Geneva: World Health Organization; 2014.

[14] Shenderovich Y, Eisner M, Mikton C, Gardner F, Liu J, Murray J (2016). Methods for conducting systematic reviews of risk factors in low- and middle-income countries. BMC Med Res Methodol.

[15] Mikton C, Power M, Raleva M, Makoae M, Al Eissa M, Cheah I, Cardia N, Choo C, Almuneef M (2013). The assessment of the readiness of five countries to implement child maltreatment prevention programs on a large scale. Child Abuse Negl.

[16] Murray J, Shenderovich Y, Gardner F, Mikton C, Derzon JH, Liu J, Eisner M, Tonry M (2018). Risk factors for antisocial behavior in low- and middle-income countries: a systematic review of longitudinal studies. Crime and justice: a review of research, vol 47.

[17] World Health Organization (2015). Preventing youth violence: an overview of the evidence.

[18] MacMillan HL, Wathen CN, Barlow J, Fergusson DM, Leventhal JM, Taussig HN. Interventions to prevent child maltreatment and associated impairment. Lancet. 2009;373:250–66. 10.1016/S0140-6736(08)61708-0 19056113

[19] Farrington DP, Welsh BC (2007). Saving children from a life of crime: early risk factors and effective interventions.

[20] Heckman JJ, García JL (2017). Social policy: targeting programmes effectively. Nat Hum Behav.

[21] Ward C, Sanders MR, Gardner F, Mikton C, Dawes A (2016). Preventing child maltreatment in low- and middle-income countries: Parent support programs have the potential to buffer the effects of poverty. Child Abuse Negl.

[22] Grantham-McGregor S, Cheung YB, Cueto S, Glewwe P, Richter L, Strupp B (2007). Developmental potential in the first 5 years for children in developing countries. Lancet.

[23] Piquero AR, Jennings WG, Diamond B, Farrington DP, Tremblay RE, Welsh BC, Gonzalez JMR (2016). A meta-analysis update on the effects of early family/parent training programs on antisocial behavior and delinquency. J Exp Criminol.

[24] Dale PS, Crain-Thoreson C, Notari-Syverson A, Cole K (1996). Parent-child book reading as an intervention technique for young children with language delays. Top Early Child Spec Educ.

[25] Blom-Hoffman J, O’Neil-Pirozzi TM, Cutting J (2006). Read together, talk together: the acceptability of teaching parents to use dialogic reading strategies via videotaped instruction. Psychol Sch.

[26] Whitehurst GJ, Falco FL, Lonigan CJ, Fischel JE, DeBaryshe BD, Valdez-Menchaca MC, Caulfield M (1988). Accelerating language development through picture book reading. Dev Psychol.

[27] Knerr W, Gardner F, Cluver L (2013). Improving positive parenting skills and reducing harsh and abusive parenting in low- and middle-income countries: a systematic review. Prev Sci.

[28] Wessels I, Mikton C, Ward C, Kilbane T, Alves R (2013). Preventing violence: evaluating outcomes of parenting programmes. Technical Report.

[29] Broidy LM, Nagin DS, Tremblay RE, Bates JE, Brame B, Dodge KA, Fergusson D, Horwood JL, Loeber R, Laird R (2003). Developmental trajectories of childhood disruptive behaviors and adolescent delinquency: a six-site, cross-national study. Dev Psychol.

[30] Engle PL, Black MM, Behrman JR, Cabral de Mello M, Gertler PJ, Kapiriri L, Martorell R, Young ME (2007). Strategies to avoid the loss of developmental potential in more than 200 million children in the developing world. Lancet.

[31] Cooper PJ, Vally Z, Cooper H, Radford T, Sharples A, Tomlinson M, Murray L (2014). Promoting mother-infant book sharing and infant attention and language development in an impoverished South African population: a pilot study. Early Childhood Educ J.

[32] Vally Z, Murray L, Tomlinson M, Cooper PJ (2015). The impact of dialogic book-sharing training on infant language and attention: a randomized controlled trial in a deprived South African community. J Child Psychol Psychiatry.

[33] Murray L, De Pascalis L, Tomlinson M, Vally Z, Dadomo H, MacLachlan B, Woodward C, Cooper PJ (2016). Randomized controlled trial of a book-sharing intervention in a deprived South African community: effects on carer-infant interactions, and their relation to infant cognitive and socioemotional outcome. J Child Psychol Psychiatry.

[34] Silva J (2007). Parents Raising Safe Kids: ACT 8-Week Program for Parents.

[35] Hallal PC, Bertoldi AD, Domingues MR, MFd S, Demarco FF, da Silva ICM, Barros FC, Victora CG, Bassani DG. Cohort profile: The 2015 Pelotas (Brazil) Birth Cohort Study. Int J Epidemiol. 2018;47(4): 1048–1048h. 10.1093/ije/dyx219. 10.1093/ije/dyx219 PMC6124621 29126133

[36] Santos IS, Barros AJ, Matijasevich A, Domingues MR, Barros FC, Victora CG (2011). Cohort profile: The 2004 Pelotas (Brazil) Birth Cohort Study. Int J Epidemiol.

[37] Victora CG, Barros FC (2006). Cohort profile: The 1982 Pelotas (Brazil) Birth Cohort Study. Int J Epidemiol.

[38] Victora CG, Hallal PC, Araújo CL, Menezes AM, Wells JC, Barros FC (2008). Cohort profile: The 1993 Pelotas (Brazil) Birth Cohort Study. Int J Epidemiol.

[39] Côté SM, Boivin M, Nagin DS (2007). The role of maternal education and nonmaternal care services in the prevention of children's physical aggression problems. Arch Gen Psychiatry.

[40] Murray E, Fernandes M, Newton CRJ, Abubakar A, Kennedy SH, Villar J, Stein A (2018). Evaluation of the INTERGROWTH-21st Neurodevelopment Assessment (INTER-NDA) in 2 year-old children. PLoS One.

[41] Reyno SM, McGrath PJ (2006). Predictors of parent training efficacy for child externalizing behavior problems—a meta-analytic review. J Child Psychol Psychiatry.

[42] Thomas R, Zimmer-Gembeck MJ (2007). Behavioral outcomes of parent-child interaction therapy and Triple P—Positive Parenting Program: a review and meta-analysis. J Abnorm Child Psychol.

[43] Piquero AR, Farrington DP, Welsh BC, Tremblay R, Jennings WG (2009). Effects of early family/parent training programs on antisocial behavior and delinquency. J Exp Criminol.

[44] Mol SE, Bus AG, de Jong MT, Smeets DJH (2008). Added value of dialogic parent-child book readings: a meta-analysis. Early Educ Dev.

[45] Weisleder A, Mazzuchelli DSR, Lopez AS, Neto WD, Cates CB, Gonçalves HA, Fonseca RP, Oliveira J, Mendelsohn AL. Reading aloud and child development: a cluster-randomized trial in Brazil. Pediatrics. 2018. 141(1). 10.1542/peds.2017-0723. 10.1542/peds.2017-0723 PMC5744270 29284645

[46] Knox M, Burkhart K, Cromly A (2013). Supporting positive parenting in community health centers: The ACT Raising Safe Kids Program. J Community Psychol.

[47] Portwood SG, Lambert RG, Abrams LP, Nelson EB (2011). An evaluation of the Adults and Children Together (ACT) Against Violence Parents Raising Safe Kids program. J Prim Prev.

[48] Knox M, Burkhart K, Howe T (2011). Effects of the ACT Raising Safe Kids Parenting Program on children's externalizing problems. Fam Relat.

[49] Knox M, Burkhart K (2014). A multi-site study of the ACT Raising Safe Kids Program: predictors of outcomes and attrition. Child Youth Serv Rev.

[50] Howe TR, Knox M, Altafim ERP, Linhares MBM, Nishizawa N, Fu TJ, Camargo APL, Ormeno GIR, Marques T, Barrios L, Pereira AI (2017). International child abuse prevention: insights from ACT Raising Safe Kids. Child Adolesc Mental Health.

[51] World Health Organization (2016). INSPIRE: seven strategies for ending violence against children.

[52] Altafim ERP, Pedro MEA, Linhares MBM (2016). Effectiveness of ACT Raising Safe Kids Parenting Program in a developing country. Child Youth Serv Rev.

[53] Pedro MEA, Altafim ERP, Linhares MBM (2017). ACT Raising Safe Kids Program to promote positive maternal parenting practices in different socioeconomic contexts. Psychosoc Interv.

[54] Silva J (2011). Programa ACT para Educar Criancas em Ambientes Seguros, Manual do Facilitador e Guia de Avaliacão [ACT Programme to Educate Children in Safe Environments: Facilitator Manual and Guide for Evaluation].

[55] Achenbach TM, Rescorla LA (2000). Manual for the ASEBA Preschool Forms and Profiles.

[56] Kochanska G, Aksan N (1995). Mother-child mutually positive affect, the quality of child compliance to requests and prohibitions, and maternal control as correlates of early internalization. Child Dev.

[57] Pereira M, Negrão M, Soares I, Mesman J (2014). Decreasing harsh discipline in mothers at risk for maltreatment: a randomized control trial. Infant Ment Health J.

[58] Sanders MR, Morawska A, Haslam DM, Filus A, Fletcher R (2014). Parenting and Family Adjustment Scales (PAFAS): validation of a Brief Parent-Report Measure for use in assessment of parenting skills and family relationships. Child Psychiatry Hum Dev.

[59] Deater-Deckard K, Lansford JE, Dodge KA, Pettit GS, Bates JE (2003). The development of attitudes about physical punishment: an 8-year longitudinal study. J Fam Psychol.

[60] Luft CDB, Sanches SO, Mazo GZ, Andrade A (2007). Versão brasileira da Escala de Estresse Percebido: tradução e validação para idosos. Rev Saude Publica.

[61] Staufenbiel SM, Penninx BW, Spijker AT, Elzinga BM, van Rossum EF (2013). Hair cortisol, stress exposure, and mental health in humans: a systematic review. Psychoneuroendocrinology.

[62] Abell JG, Stalder T, Ferrie JE, Shipley MJ, Kirschbaum C, Kivimaki M, Kumari M (2016). Assessing cortisol from hair samples in a large observational cohort: The Whitehall II Study. Psychoneuroendocrinology.

[63] Simmons JG, Badcock PB, Whittle SL, Byrne ML, Mundy L, Patton GC, Olsson CA, Allen NB (2016). The lifetime experience of traumatic events is associated with hair cortisol concentrations in community-based children. Psychoneuroendocrinology.

[64] Noppe G, Van Rossum EFC, Koper JW, Manenschijn L, Bruining GJ, de Rijke YB, van den Akker ELT (2014). Validation and reference ranges of hair cortisol measurement in healthy children. Horm Res Paediatr.

[65] Boeckel MG, Viola TW, Daruy-Filho L, Martinez M, Grassi-Oliveira R (2017). Intimate partner violence is associated with increased maternal hair cortisol in mother-child dyads. Compr Psychiatry.

[66] Goodman R (2001). Psychometric properties of the Strengths and Difficulties Questionnaire. J Am Acad Child Adolesc Psychiatry.

[67] Howard SJ, Melhuish E (2017). An Early Years Toolbox for assessing early executive function, language, self-regulation, and social development: validity, reliability, and preliminary norms. J Psychoeduc Assess.

[68] Denham SA, Bassett HH, Way E, Kalb S, Warren-Khot H, Zinsser K (2014). ‘How would you feel? What would you do?’ Development and underpinnings of preschoolers’ social onformation processing. J Res Child Educ.

[69] Rieffe C, Ketelaar L, Wiefferink CH (2010). Assessing empathy in young children: construction and validation of an Empathy Questionnaire (EmQue). Personal Individ Differ.

[70] Buttelmann D, Carpenter M, Tomasello M (2009). Eighteen-month-old infants show false belief understanding in an active helping paradigm. Cognition.

[71] Benenson JF, Pascoe J, Radmore N (2007). Children's altruistic behavior in the dictator game. Evol Hum Behav.

[72] Baron-Cohen S, Leslie AM, Frith U (1985). Does the autistic child have a ‘theory of mind’ ?. Cognition.

[73] Abell F, Happé F, Frith U (2000). Do triangles play tricks? Attribution of mental states to animated shapes in normal and abnormal development. Cogn Dev.

[74] Tremblay RE (2010). Developmental origins of disruptive behaviour problems: the ‘original sin’ hypothesis, epigenetics and their consequences for prevention. J Child Psychol Psychiatry.

